# Dual pathogen valvular endocarditis in a case of severe aortic aneurysm

**DOI:** 10.4322/acr.2021.381

**Published:** 2022-05-05

**Authors:** Meghna Yadav, Rohit Tewari, Tathagata Chatterjee

**Affiliations:** 1 Base Hospital, Department of Pathology, New Delhi, India; 2 Army Hospital, Department of Laboratory Science & Molecular Medicine, New Delhi, India

**Keywords:** Endocarditis, Acinetobacter baumannii, Aneurysm, Aspergillus

## Abstract

Infective endocarditis (IE) is the heart valve or endocardium infection. We report a rare case of polymicrobial endocarditis, namely invasive *Aspergillus spp* and *Acinetobacter baumannii,* in a 36-year-old male with a medical history of degenerative disease of the aorta with abdominal aortic and ascending aortic aneurysms with a fulminant clinical course and fatal outcome. The treatment was challenging due to multiple comorbidities. The autopsy revealed dual pathogen endocarditis due to *Acinetobacter baumannii* sepsis and invasive *Aspergillus spp* mycosis. This report emphasizes that polymicrobial endocarditis (PE) is an infrequent finding with a poor prognosis requiring high clinical suspicion.

## INTRODUCTION

Infective endocarditis is an uncommon, potentially lethal infectious disease with multiple etiologies. Even with appropriate antibiotic usage and surgical intervention, IE is still a life-threatening infection with high mortality rates. Most infections are bacterial, mostly Gram-positive cocci like *Staphylococcus aureus* and *Streptococci spp*. A single causative pathogen is established in almost 90% of the cases.^1^ However, polymicrobial endocarditis (PE) is quite uncommon, ranging from 1% to 6.8% and is associated with poor prognosis.^2^

*Acinetobacter baumannii* is an emerging nosocomial infection harboring multidrug resistance properties and high mortality and morbidity risk. Invasive medical procedures and widespread use of broad-spectrum antibiotics increase the risk of this Gram-negative coccobacillus.^3^ The clinical course of native valve endocarditis due to *Acinetobacter* spp. is often acute onset and aggressive. Fungal endocarditis is one of the fatal forms of IE, accounting for less than 10% of all cases. Aspergillus endocarditis (AE) is often severe, occurring in almost 25% of all cases of fungal endocarditis.^4^ The co-existence of bacterial and fungal etiological agents is an unusual occurrence, which prompted us to report the case.

## CASE REPORT

A 36-year-old male with a medical history of advanced atherosclerosis of the aorta with aneurysm of the right and left sinus of Valsalva, abdominal aortic aneurysm, and renal artery stenosis (post stenting) was admitted with chief complaints of acute-onset high-grade fever and pedal edema. The patient had undergone aortic root replacement and coronary artery bypass graft (CABG) two months prior. His medical history included the headache workup and Grade-IV hypertensive retinopathy, right renal artery stenosis at 11 mm from the origin for which he underwent renal artery stenting 11 years prior. The patient also had a 12.5 cm abdominal aortic aneurysm in the suprarenal abdominal aorta, for which endovascular aneurysm repair (EVAR) with visceral debranching and polytetrafluoroethyelene (PTFE) graft was done eight years back. He also underwent Bentall’s procedure four months prior to current admission for sinus of Valsalva with a massive aneurysm with thrombosis, which was detected on imaging on follow-up.

On admission, his blood pressure was 120/88 mmHg, heart rate was 142/min, the temperature was 38,3 °C, pallor. He was pale and pedal edema was present. The patient was diagnosed with acute left ventricular failure with suspected infective endocarditis in view of prior surgeries, fever, and edema. He was started on ceftriaxone, vancomycin, and gentamycin. Fever persisted, and he developed oliguria, unresponsive to diuretics, progressive breathlessness, hypotension; and had a sudden cardiac arrest on the 7^th^ day of admission and died.

The laboratory work-up showed hemoglobin of 7.8 g/dL (reference value [RV]: 12-14 g/dL), leukocytes of 9,670/ mm3 (RV; 4.4-11.3 × 103/mm3) without a shift to the left, and platelets of 156,000/mm3 (RV; 150-400 × 103/mm3), creatinine of 1.3 mg/dL (RV: 0.4-1.3 mg/dL), and normal electrolytes. Bedside 2D echocardiogram showed left ventricular function of 15-20% with moderate aortic regurgitation. Paired blood culture revealed growth of *Acinetobacter* species.

## AUTOPSY FINDINGS

The external examination showed the deceased to be averagely built and icteric, with moderate edema of the lower limbs. A linear scar over the thorax and abdomen was noted.

**Figure 1:**
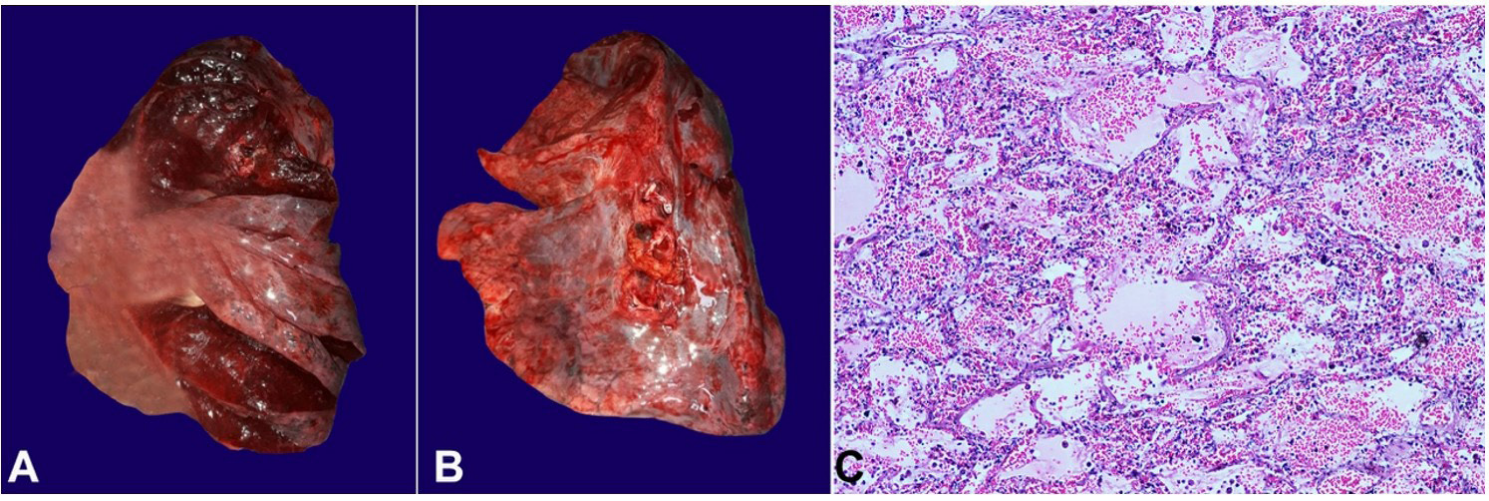
**A** and **B –** Gross view of the unfixed lung showing boggy and edematous surface with exudation of frothy fluid; **C** – Photomicrograph of lung at low magnification showing diffuse alveolar and interstitial edema with focal areas exhibiting intra-alveolar hemorrhage (H&E).

The thoracic cavity contained 1000 mL of serous fluid. The right lung weighed 730 gm (mean reference value [MRV]: 450 g) and the left lung 680 gm (MRV: 375 gm), which were boggy, markedly edematous, and adherent to the pleura.150 ml of serous pericardial effusion was present. The cut surface showed exudation of frothy fluid. No pale area, focal lesion, or abscess was noted. Although the lungs were extensively sectioned, no infectious or inflammatory lesion was detected on gross (Figure 1). The histological examination showed diffuse alveolar and interstitial edema with focal areas exhibiting intra-alveolar hemorrhage. The pericardial surface was rough, shaggy, and had adhesions. Cardiovascular examination showed an enlarged heart weighing 550 gm [MRV: 275 gm). The epicardial fat was preserved. There was right ventricular hypertrophy (1.4 cm (mRR; 0,38 cm) and left ventricular wall was 2.5 cm (mRR; 1,15 cm) thick. The right and left ventricles with the valve leaflets, papillary muscles, and chordae tendinea appeared thickened on gross examination.

**Figure 2:**
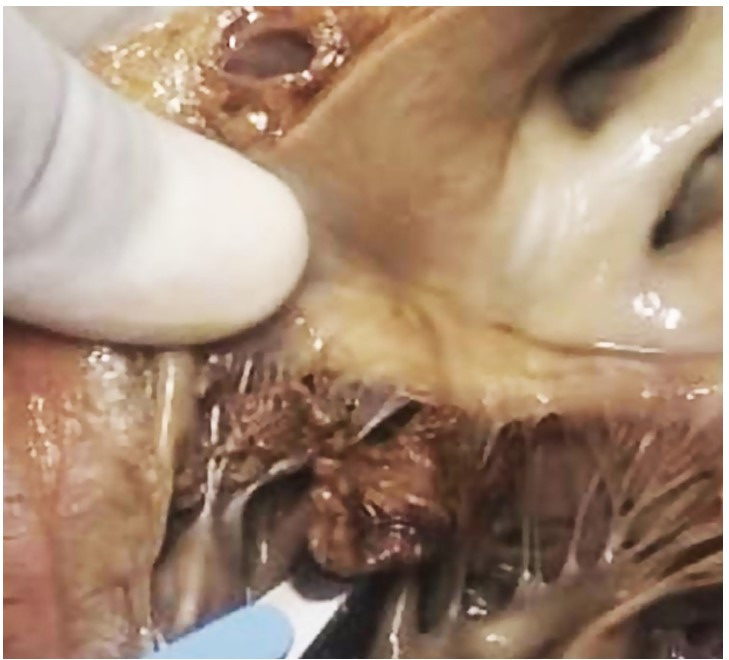
Gross view of the mitral valve showing large vegetation measuring 2.5x1.9x.0.6 cm. The mitral valve showed tissue corrosion on the posterior leaflet and anterior commissure.

A single fungating friable vegetation was seen on the mitral valve measuring 1.5x1.1x.0.6 cm along the closure of cusps (Figure 2). The mitral valve showed tissue corrosion on the posterior leaflet and anterior commissure. The heart was adherent with the pericardium. The coronaries showed CABG grafts with no evidence of atherosclerosis.

**Figure 3:**
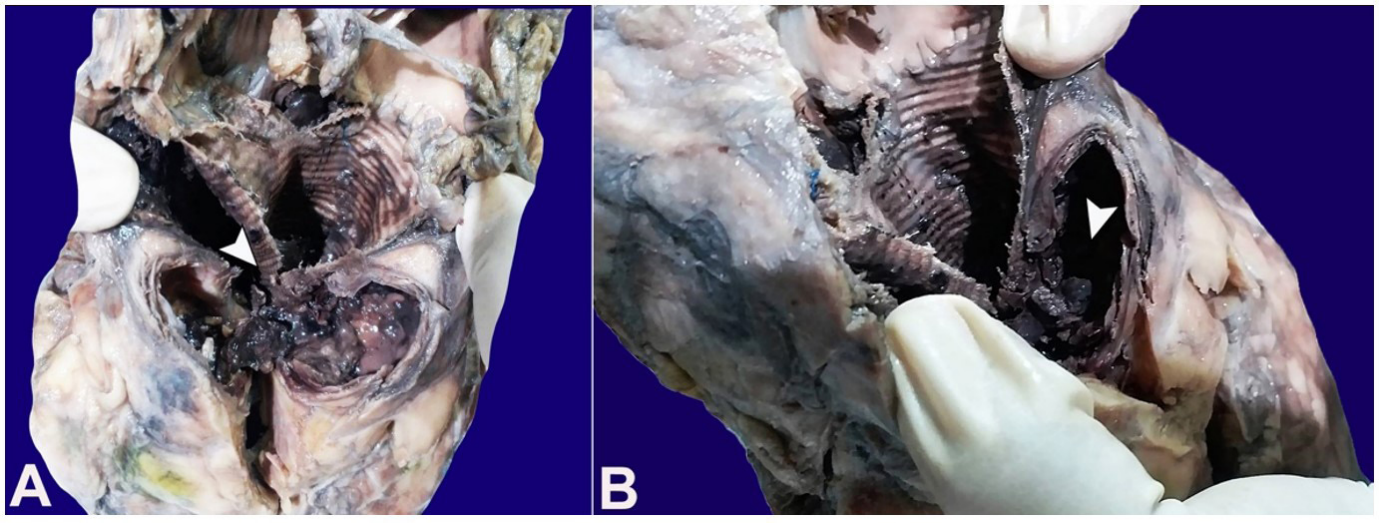
**A** and **B** – Gross view of massive aneurysmal dilation of the sinuses of Valsalva with blood clots in the lumen (arrowheads). The surrounding tissue showed purulent material.

**Figure 4:**
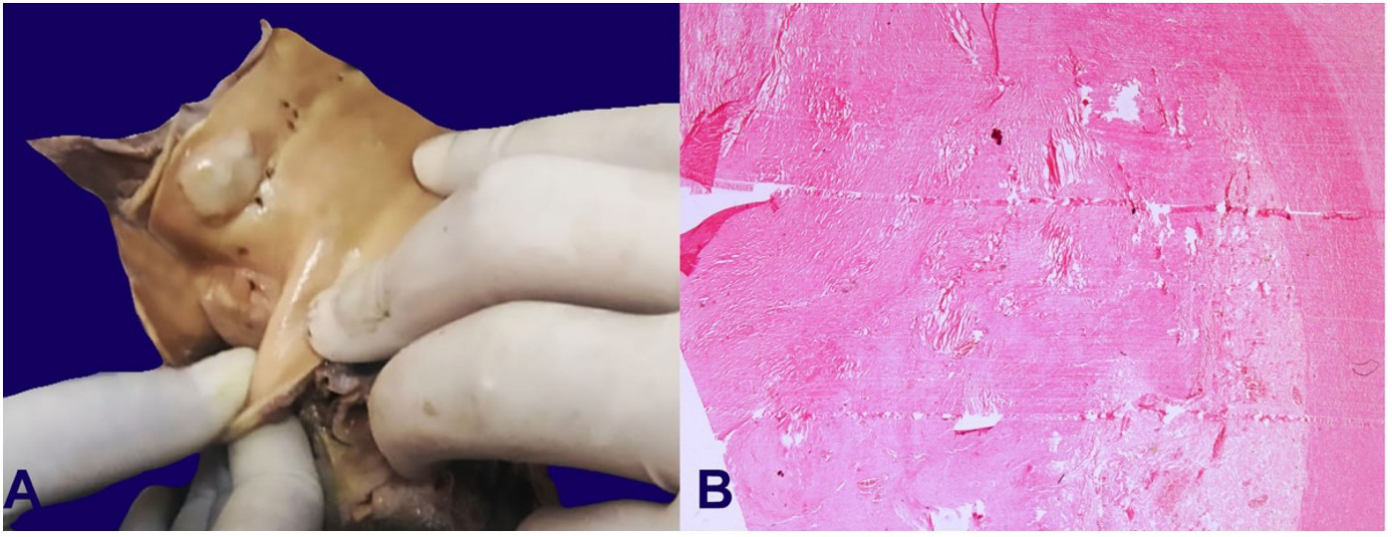
**A** – Gross view of ascending aorta shows nodular lesions on the surface; **B** – Photomicrograph of aorta shows elastic tissue fragmentation and areas devoid of elastic tissue. No foci of inflammation, plasma cell clusters, granulomas, or giant cells were found.

**Figure 5:**
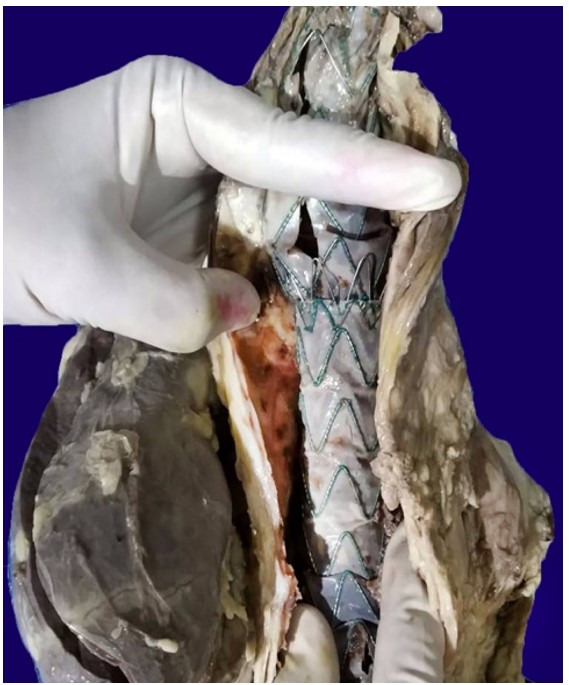
The ascending aorta shows thickened nodular lesions on the luminal surface measuring 1.5 cm each with local ulceration; also note the large abdominal aortic aneurysm measuring 10 cm in extension and 5.0 cm in diameter filled in by an aortoiliac prosthesis, which was extensively thrombosed above the origin of the renal arteries.

A massive aneurysmal dilation of the sinuses of Valsalva with blood clots in the lumen was noted (Figures 3A, and 3B). The ascending aorta showed thickened nodular lesions on the luminal surface measuring 1.5 cm each with local ulceration (Figure 4). Large abdominal aortic aneurysm measuring 10 cm in extension and 5.0 cm in diameter filled in by an aortoiliac prosthesis, which was extensively thrombosed, was present above the origin of the renal arteries (Figure 5). The abdominal aorta was thickened.

The left kidney was enlarged and weighed 210 gm [ MRV: 170 gm], the external surface was bosselated and showed fetal lobulations. The capsule could be easily stripped off. The cut surface appeared congested, but cortico-medullary demarcation could be made out easily. A wedge-shaped subcapsular pale area was seen at the corticomedullary junction measuring 2.1x 1.4x 0.4 cm. The right kidney showed a pale subcapsular area that was wedge-shaped. Perirenal fat appeared normal.

**Figure 6:**
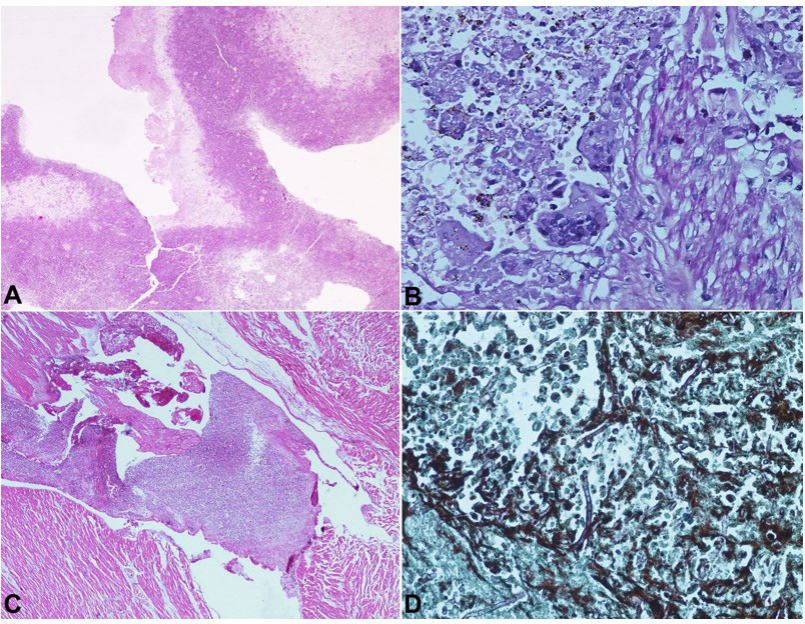
Photomicrographs of the mitral valve vegetation. **A** – Low magnification showing destructive inflammatory infiltrate comprised predominantly of neutrophils, few lymphoplasmacytic cells and macrophages (PAS, 20 x); **B** – Photomicrograph of myocardial abscess in left ventricular wall showing blood, fibrin clot along with colonies of embedded bacterial organisms. Numerous broad septate hyphae suggestive of *Aspergillus* sp eliciting giant cell reaction and chronic inflammatory infiltrate. Histomorphological features was consistent with fresh vegetation composed of fibrin, granulation tissue and neutrophils (PAS, 40 x); **C** – Photomicrographs of the mitral valve vegetation showing numerous bacterial colonies infiltrating the heart muscle (H&E, 20 x); **D** – Photomicrographs of the mitral valve vegetation. Grocott stain shows acute angle branching hyphae with frequent septations morphologically consistent with *Aspergillus* sp (40 x).

On microscopic examination, sections from mitral valve vegetation and tricuspid valve showed destructive inflammatory infiltrate comprised predominantly of neutrophils, few lymphoplasmacytic cells, and macrophages (Figure 6A). Histomorphological features were consistent with fresh vegetation composed of fibrin, granulation tissue, and neutrophils (Figure 6B). Numerous bacterial colonies of Gram-negative bacilli, and septate fungal hyphae morphologically consistent with *Aspergillus* sp, were also noted.

Sections from the aortic valve at the overlying implant and thrombi in the sinus showed blood, fibrin clot along with colonies of embedded bacterial organisms (Figure 6C). Numerous broad septate hyphae suggest *Aspergillus* sp eliciting giant cell reaction and chronic inflammatory infiltrate (Figure 5D). Giant cell reaction was also seen to foreign material. Thrombus showed areas of ossification.

The sections from the myocardium showed edema along with mild lymphomononuclear inflammatory infiltrate. No giant cells/granulomas were seen. Foci of neutrophilic microabscesses were seen in the myocardium. The sections from the right and left coronary arteries beyond stented areas showed patent vessels with mild intimal fibrosis.

**Figure 7:**
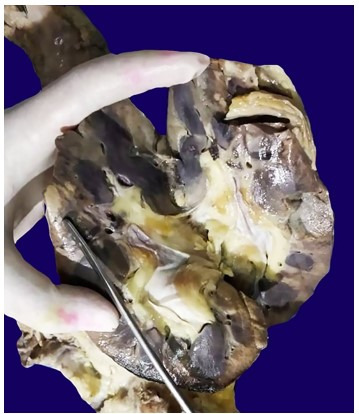
Gross view of the enlarged left kidney, weighing 210 gm (MRV: 170 g). Note a wedge-shaped subcapsular pale area indicating septic infarct measuring 2.1x1.4x 0.4 cm.

The sections from nodular lesions on the endothelial surface of ascending aorta showed a fibrous stage of atherosclerotic plaques. The sections from the aorta showed elastic tissue fragmentation and areas devoid of elastic tissue. Accumulation of basophilic material was noted in the wall. There were no foci of inflammation, plasma cell clusters, granulomas of giant cells seen. The sections from the right kidney showed features of infarcts in the form of widespread coagulative necrosis with preserved ghost lines (Figure 7).

**Figure 8:**
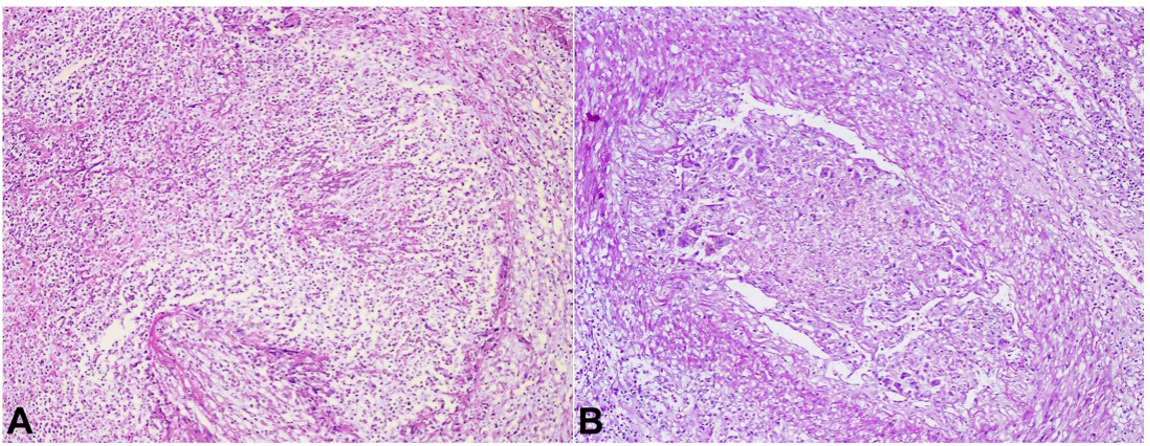
Photomicrographs of the kidney. **A** – showing features of infarcts in widespread coagulative necrosis (preserved ghost lines) (H&E 20 x); **B** – Left renal septic emboli with fungal hyphae. Thrombi was seen in the vessels at the apex of the infarct. The thrombi showed blood, fibrin and septate hyphae consistent with *Aspergillus* spp. (PAS, 40 x).

Thrombi were seen in the vessels at the apex of the infarct. The thrombi showed blood fibrin, and septate hyphae consistent with *Aspergillus* sp (Figure 8A and 8B). The remaining study of the kidney showed focally sclerotic glomeruli, interstitial edema, and scattered lymphomononuclear infiltrate. Tubules in the non-necrotic areas show features of acute tubular necrosis.

## DISCUSSION

The incidence of polymicrobial endocarditis (PE) is increasing. PE is associated with more aggressive clinical presentation and fatal outcomes than monomicrobial presentation.^5^ PE has been described most frequently in intravenous drug abusers, patients with valvular prosthesis, compromised immune status, diabetes and end-stage renal disease.^6^ Accordingly, multiple factors predisposed our patient to PE. In this case the patient was submitted to cardiac manipulation, extracorporeal circulation, catheterization and may have used long polymicrobial regimens. Despite not considering him a typical immunosuppressed host, his clinical history may have deteriorated his immune system. These factors may have set the patient at risk of t developing PE.

Dual pathogen endocarditis poses a diagnostic challenge, notably when pathogens are recoverable from different specimens. We established bacterial pathogenicity based on the isolation of *Acinetobacter* species from paired blood cultures. In our case, the diagnosis of invasive aspergillosis was only confirmed on histomorphology as blood cultures for yeast were negative. The diagnosis of fungal endocarditis is intricate due to the poor yield from blood cultures, which are only positive in less than 50% of cases.^4^ There could have been a possibility that the Aspergillus infection evolved insidiously from the time of surgery and led to hematogenous infection of a more fulminant course.

This case highlights the importance of a rapid diagnosis coupled with an appropriate treatment to prevent the fatal outcome in a young male with severe underlying atherosclerosis.

There are only a few published case reports of PE with bacterial and fungal etiology. Published reports include PE with *Streptococcus sanguis* and *Phialemonium. aff curvatum*, *Listeria monocytogenes* and *Acremonium* spp, *Burkholderia cepacian* and *Aspergillus flavus* and PE due to *Candida tropicalis* with *Staphylococcus aureus*.^7^^,^^8^^,^^9^^,^^10^ This is, to our knowledge, the first case of PE involving *Acinetobacter baumannii* and *Aspergillus spp.*

The standard care in such cases includes a combination of medical and surgical therapy. In the index case, the patient was given antibiotics; however, no antifungal was added. The result of galactomannan was awaited and turned out positive. Serological assay for detecting galactomannan, a molecule found in the cell wall of *Aspergillus spp* can help diagnose invasive aspergillosis infection in humans.^11^ The addition of Voriconazole can be a valuable step when suspecting fungal endocarditis.^12^

*Acinetobacter* species are becoming increasingly important as a cause of healthcare-associated infections like pneumonia, bloodstream infections, and urinary tract infection. Acinetobacter is ubiquitous pleomorphic, Gram-negative coccobacilli capable of adhering to surfaces and forming biofilms. As an opportunistic pathogen, *Acinetobacter* species mainly leads to nosocomial infections, and its multidrug resistance properties have gained more and more attention. There are numerous risk factors associated with *Acinetobacter* spp. bacteremia such as invasive procedures, open-heart surgery, dental work, and intravenous drug abuse. Most likely, in our case, the infection is secondary to multiple invasive procedures. Menon and colleagues^13^ also reported a similar case of IE caused by *Acinetobacter* spp. in a 27-year-old male occurring within a month of post-surgical repair of a ruptured aneurysm of the sinus of Valsalva.

Aspergillus endocarditis is second only to candida endocarditis and is mostly culture-negative emphasizing the challenging diagnosis and its poor prognosis. Histopathological evidence of tissue invasion is needed for establishing a diagnosis of invasive Aspergillus.^9^ Predisposing risk factors for fungal endocarditis includes prior cardiac surgery or the presence of a prosthetic valve, intravenous drug use, immunocompromised states and indwelling vascular lines.^14^

In the last decade, invasive medical and diagnostic procedures have led to a hike in the incidence of healthcare-associated IE, accounting for 10-34% of all IE cases.^15^ It is a growing health concern with high mortality. Various studies have described the high incidence of Gram-negative and fungi infection as the etiological agent in healthcare-associated IE.^16^ Clinicians must be aware of the potential risk of endocarditis in the context of previous vascular manipulation and predisposing factors and maintain strict infection control and contact precaution policies during manipulation of vascular lines.

## CONCLUSION

IE is still associated with high morbidity and mortality. Dual pathogen endocarditis is a rare disease entity and poses a diagnostic challenge. This case report also calls attention to the principal aim of the clinical autopsy, which is the improvement of the care quality and to yield insight into the pathological process. It is imperative that clinicians maintain a high index of suspicion and should be aware of the association of hospital-derived pathogens such as *Acinetobacter* spp and invasive *Aspergillus* with prosthetic valve endocarditis occurring long after the initial cardiothoracic procedure.
